# MiR-223-3p attenuates the migration and invasion of NSCLC cells by regulating NLRP3

**DOI:** 10.3389/fonc.2022.985962

**Published:** 2022-10-06

**Authors:** Shasha Zhu, Xiangbing Kong, Mengru Song, Mingyang Chi, Yitong Liu, Peng Zhang, Qiao Zhang, Pingping Shang, Feifei Feng

**Affiliations:** ^1^ College of Public Health, Zhengzhou University, Zhengzhou, China; ^2^ College of Medicine, Zhengzhou University, Zhengzhou, China; ^3^ College of Public Health, University of Southern California, Los Angeles, CA, United States; ^4^ Department of Bone and Soft Tissue Cancer, The Affiliated Cancer Hospital of Zhengzhou University (Henan Cancer Hospital), Zhengzhou, China; ^5^ Key Laboratory of Tobacco Chemistry, Zhengzhou Tobacco Research Institute, CNTC, Zhengzhou, China

**Keywords:** miR-223-3p, NLRP3, NSCLC, invasion, migration

## Abstract

Lung cancer is the malignant tumor with high invasion and metastasis, which seriously threatens public health. Previous study showed that NLRP3 could promote the occurrence of lung tumors in B(a)P-induced mice. MicroRNAs are closely related to the progression and metastasis of lung cancer by regulating target genes. However, which miRNAs affect the migration and invasion of lung cancer cells through regulating NLRP3 remains poorly defined. In this study, the miRNAs targeting NLRP3 were selected from TargetScan and miRDB database and finally miR-223-3p was chosen due to the consistent expression in both A549 and H520 cells. Then, the migration and invasion of lung cancer cells were detected with miR-223-3p mimic and inhibitor using Transwell assay, at the same time the expression of NLRP3, cleaved caspase-1, IL-1β and IL-18 was determined using Western Blot and immunohistochemistry assay. Our data demonstrated that miR-223-3p was upregulated in both A549 and H520 cells. Furthermore, the migration and invasion of A549 and H520 cells were promoted after inhibiting miR-223-3p. Besides, the levels of NLRP3, cleaved caspase-1, IL-1β and IL-18 were increased in the two lung cancer cells. And the corresponding results were contrary in miR-223-3p mimic group. Taken together, miR-223-3p attenuates the migration and invasion of NSCLC cells by regulating NLRP3, which provides evidence for the prevention and targeted treatment of NSCLC.

## Introduction

Lung cancer is the leading cause of cancer-related death worldwide. According to *Global Cancer Statistics 2020*, lung cancer ranked first on the mortality and ranked second on the incidence among all the top 10 most common cancers, which is severely threatening to human health (1). Among various kinds of lung cancer, there are approximately 80-85% are non-small cell lung cancer (NSCLC), including adenocarcinoma and squamous cell carcinoma (2). Recently, though a large number of studies about lung cancer have been carried out all over the world, the prognosis of lung cancer is still not optimistic and the case fatality rate is still high. One of the main reasons is that most lung tumors have invaded and migrated before they were first diagnosed by available medical methods (3). Therefore, it is vital to explore the mechanism of the development of NSCLC for effective therapy.

Chronic inflammation is closely related to the occurrence and progression of lung cancer (4). The pattern recognition receptors of the leucine-rich nucleotide binding domain receptor family (NLR) play an important role in inflammation caused by innate immune system (5). NLRP3 is an important member of NLRs family. It forms the NLRP3 inflammasome with pro-caspase-1 and ASC (apoptosis-associated speck-like protein containing a carboxy-terminal CARD) (6). After NLRP3 inflammasome being activated, pro-caspase-1 was cleaved to cleaved caspase-1, and subsequently, the cleaved caspase-1 matured pro-IL-1β and pro-IL-18 into IL-1β and IL-18. NLRP3 inflammasome has anti-cancer effects on colon cancer and pro-cancer effects on gastric cancer and lung cancer (7–9). Its downstream product, IL-18, can increase the cytotoxicity of NK cells, suggesting that NLRP3 inflammatory bodies have anti-cancer effects, and IL-1β and IL-18 have also been proven to inhibit anti-metastasis and immune surveillance mediated by NK cells and T cells to promote the occurrence and development of cancer (10). Our previous study showed that NLRP3 inflammasome participated in the tumorigenesis of lung cancer and deletion of NLRP3 gene could inhibit the occurrence of lung tumors in mice induced by B(a)P or B(a)P combined with LPS (11). Moreover, NLRP3 could be regulated by microRNAs.

microRNA (miRNA) is a class of small noncoding RNA which negatively regulate the gene translation or degrade mRNAs by binding to the 3′-untranslated region (3′-UTR) of target genes (12). Studies have shown that miRNAs are overexpressed in malignant tumors such as breast cancer, lung cancer, gastric cancer and prostate cancer which indicates that there is an intimate relationship between tumorigenesis and abnormal expression of miRNAs (13–16). In particular, the abnormal expression of miRNA is closely related to the diagnosis, progression, metastasis, treatment and prognosis of lung cancer (17). A previous study demonstrated that miRNA inhibited the proliferation and migration of malignant glioma cells, human gastric cancer cells and oral squamous carcinoma cells by regulating NLRP3 (18–20). In addition, it has been reported that the activation of NLRP3 inflammasome can promote the proliferation, migration and invasion of A549 cells (21). However, which miRNAs affect the migration and invasion of lung cancer cells through regulating NLRP3 still remain unknown.

In this study, we aimed to select the miRNAs targeting NLRP3 from TargetScan and miRDB Online Database and finally choose the most suitable miRNA for the further experiments due to the expression in lung cancer cells. Then the selected miRNA was overexpressed or inhibited in lung adenocarcinoma cells (A549) and lung squamous carcinoma cells (H520), respectively. After that, the migration and invasion ability of the two cells were measured, and the protein expression of NLRP3, cleaved caspase-1, IL-1β and IL-18 were detected to reflect the expression and activation of NLRP3 inflammasome. This study will provide clues for the regulatory mechanism of the migration and invasion of NSCLC, and finally promote the prevention and targeted treatment of NSCLC.

## Materials and methods

### miRNA selection

The aimed miRNAs were selected from the TargetScan and miRDB Online Database which directly target at NLRP3 by the following two standards: 1) TargetScan database conservative target probability (P_CT_)≧0.3 or miRDB database prediction target score>85; 2) The selected miRNAs meet the homologous condition of human and mouse. The selected miRNAs targeting NLRP3 were miR-223-3p, miR-22-3p and miR-1305.

### Cell culture

Human lung adenocarcinoma cell line (A549), human lung squamous carcinoma cell line (H520) and normal human bronchial epithelial cell (BEAS-2B) were purchased from Shanghai Institute of Biochemistry and Cell Biology, CAS. All of the cell lines were cultured in RPMI 1640 medium (Solarbio, Beijing, China) containing 10% fetal bovine serum (FBS; Solarbio, Beijing, China), under a 5% CO_2_ atmosphere at 37°C.

### Quantitative real-time polymerase chain reaction

The total RNA from A549 and H520 cells were extracted with Trizol Reagent (Invitrogen, USA) according to the operation instructions. cDNA was synthesized with a reverse transcription kit (TIANGEN BIOTECH, China), and then the quantitative real-time polymerase chain reaction was conducted in a 7500 Fast Real-time PCR System. The primer sequences in this study were as follows: miR-22–3p, 5′-AACAGTGAAGCTGCCAGTTGAA3′ (reverse); miR-223–3p, 5′-CGCTGTCAGTTTGT-CAAATACCCCA-3′ (reverse); miR-1305, 5′-GCCGCGCGTTTTCAACTCTAATGGGAG-3′ (reverse); U6, 5′-CTCGCTTCGGCAGCACA-3′ (forward) and 5′-AACGCTTCACGAATTTGCGT-3′ (reverse). The data obtained was analyzed as 2^-△△Ct^. Each experiment was carried out in triplicate.

### Cell transfection

miR-223-3p mimic and miR-223-3p inhibitor (Guangzhou Ruibo Biotechnology Co, Ltd) were all given to A549 and H520 cells respectively to overexpress miR-223-3p or suppress the function of miR-223-3p. miR-223-3p inhibitor control was inhibitor NC and miR-223-3p mimic control was mimic NC. The sequences were as follows: miR-223-3p inhibitor: 5′-UGGGGUAUUUGACAAACUGACA-3′; miR-223-3p mimic: 5′-UGUCAGUUUGUCAAAUACCCCA-3′ (forward) and 5′- UGGGGUAUUUGACAAACUGACA-3′ (reverse); inhibitor NC: 5′-CAGUACUUUUGUGUAGUACAAA-3′; mimic NC: 5′-UUUGUACUACACAAAAGUACUG-3′ (forward) and 5′- CAGUACUUUUGUGUAGUACAAA -3′ (reverse). miR-223-3p inhibitor and miR-223-3p mimic were used with transfection kits (Ruibo Biotechnology Co, Ltd, China) according to the manufacturer’s instruction when the cells in the six-well plate were fused to 40–50%. The concentrations of miR-223-3p inhibitor and miR-223-3p mimic are both 100nM per well. After 6h transfection, cells are replaced with new RPMI 1640 medium. Follow-up experiments were performed when cells fused to 80%-90%.

### Cell migration and invasion assays

Matrigel matrix glue diluted in precooled serum-free medium was spread on the culture chamber with 8µm small-hole polycarbonate filter membrane, and 100 µL serum-free medium diluted A549 cells or H520 cells were inoculated; in the lower chamber, RPMI 1640 culture medium containing 10% serum of 600pL was added, each group had 6 multiple holes. After being incubated at 37°C and 5% CO_2_ for 24 hours, the culture chamber was taken out, then it was fixed with 2.5% glutaraldehyde for 15min, treated with 0.5% TritonX-100 for 3min and stained with crystal violet for 15min. Invert the culture chamber, observe and photograph it under an optical microscope (Leica, Germany) to count the average number of cells at the bottom of the filter membrane per high vision field. The cell migration experiment was carried out in the Transwell chamber without Matrigel matrix glue in the same method.

### Western blot analysis

Cells were collected, trypsinized and lysed in RIPA lysis buffer. Transfer the electrophoresed proteins to a poly (vinylidene fluoride) membrane and incubate for 2 hours at room temperature in blocking solution. Then they were incubated in the membrane overnight at 4°C in antibody solution containing primary antibody, including anti-NLRP3 antibody and caspase-1 antibody (Cell Signaling Technology, USA). After overnight incubation, second antibody goat-anti-rabbit (1:5000) were added at 37 °C for 1.5h. The membrane was washed at room temperature for 30min and then detected with Amersham Imager 600 automatic chemiluminescence gel imaging analyzer.

### Immunohistochemistry

After the cells were incubated with hydrogen peroxide for 20 min and blocked with TBST containing goat serum for 25 min at room temperature, they were incubated overnight at 4°C in primary antibody, including anti-IL-1β antibody and anti-IL-18 antibody (1:100 dilution), respectively. On the second day, the second antibody, goat anti-rabbit (1:100 dilution), were added at 37°C for 40 min. In 200 high vision field of microscope, the cell was stained in blue while the positive one was brown. We used the semi-quantitatively analysis to analyze the collected images and the results were expressed by the average optical density of the brown area.

### Statistical analysis

The data was analyzed using SPSS 23.0 software. Data was expressed as mean ± standard deviation (SD). The independent sample *t* test was used to compare the data between two groups. The difference was statistically significant when α=0.05.

## Results

### miR-223-3p was upregulated in A549 and H520 cells

**Figure 1:**
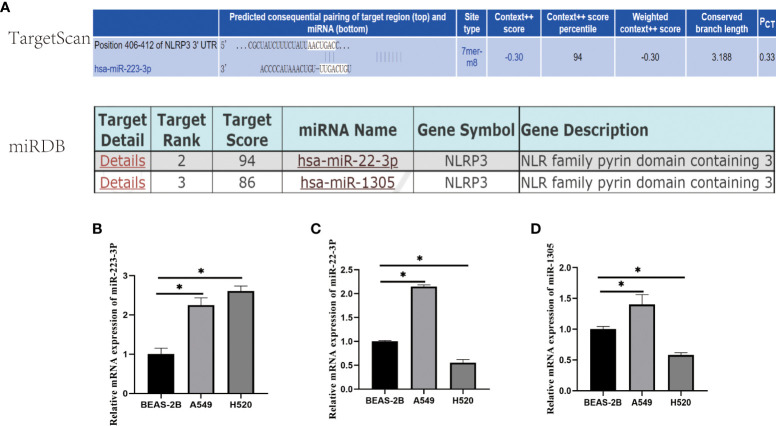
The selection and expression of miRNAs regulating NLRP3. **(A)** The target score of the selected miRNAs binding to NLRP3 gene. miR-223-3p was selected from TaargetScan Database with P_CT_≧0.3; miR-22-2p and miR-1305 were selected from miRDB Database with predicted target scores >85. **(B)** The relative expression of miR-223-3p. **(C)** The relative expression of miR-22-3p. **(D)** The relative expression of miR-1305. Data were presented as means ± SD of three independent experiments. *P<0.05.

We selected the miRNAs from the TargetScan and miRDB Online Database which directly target at NLRP3 gene, and they were miR-223-3p, miR-22-3p, and miR-1305 (**Figure 1A**). To figure out whether the miRNA above have the same influence on the progress of A549 andF H520 cells, qRT-PCR was first carried out to detect the expression in the cells. As presented in **Figure 1**, comparing to BEAS-2B, the three miRNAs above were all expressed irregularly in A549 and H520 cells. The results showed that the expression of miR-223-3p was remarkably increased in both A549 and H520 cells (**Figure 1B**); the expression of miR-22-3p and miR-1305 was increased in A549 cells while was decreased in H520 cells (**Figures 1C, D**). These results suggested that the selected miRNAs were differently dysregulated in different lung cancer cells but miR-223-3p was upregulated in both A549 and H520 cells.

### Inhibition of miR-223-3p enhanced migration and invasion of A549 and H520cells

**Figure 2:**
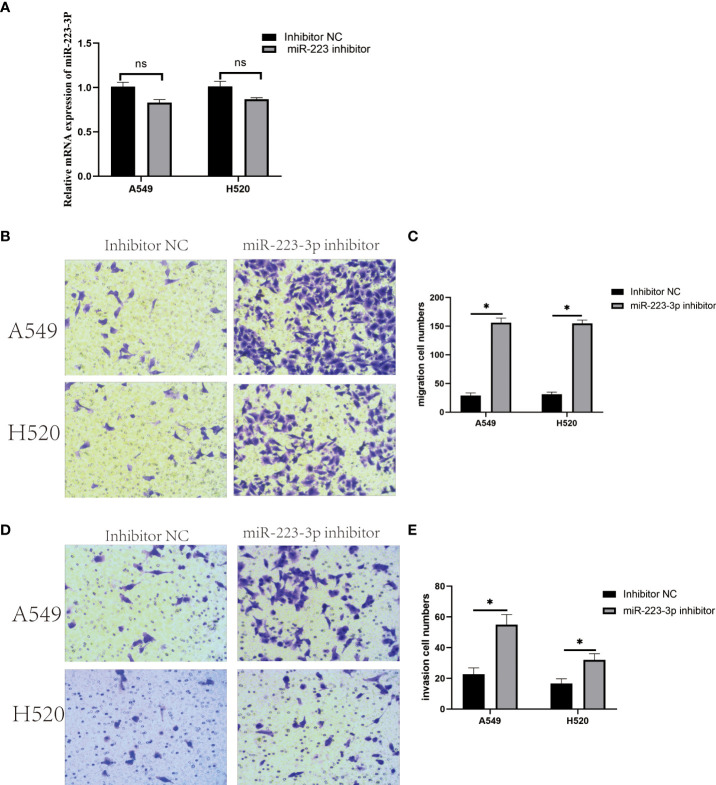
Downregulation of miR-223-3p promoted the migration and invasion of A549 and H520 cells. **(A)** The relative expression of miR-223-3p after miR-223-3p inhibitor transfection was detected by qRT-PCR. **(B)** Cells at the bottom of the filter membrane without Matrigel matrix glue were detected by Transwell assay in high vision field (200×). **(C)** Quantitative analysis of the migration cells of A549 and H520. **(D)** Cells at the bottom of the filter membrane with Matrigel matrix glue were detected by Transwell assay in high vision field (200×). **(E)** Quantitative analysis of the invasion cells of A549 and H520. Data were presented as means ± SD of three independent experiments. *P<0.05, ns: no significance.

According to the results of qRT-PCR, the irregular expression of miR-223-3p was consistent in both A549 and H520 cells, so we focused on miR-223-3p for the further experiments. The A549 and H520 cells were transfected with miR-223-3p inhibitor which could bind to miR-223-3p in the form of base complementary pairing to suppress the function of miR-223-3p so that the inhibitor groups were established successfully as **Figure 2A**. Next, the effect of miR-223-3p suppression on the migration and invasion of A549 and H520 cells was assessed by Transwell assay. When A549 and H520 cells were transfected with miR-223-3p inhibitor, the average number of cells at the bottom of the filter membrane without or with Matrigel matrix glue was significantly increased which meant that the migration (**Figures 2B, C**) and invasion (**Figures 2D, E**) of A549 and H520 cells were promoted remarkably. These results indicated that it would promote the migration and invasion of A549 and H520 cells with the inhibition of miR-223-3p.

### Inhibition of miR-223-3p promoted the expression and activation of NLRP3 inflammasome

**Figure 3:**
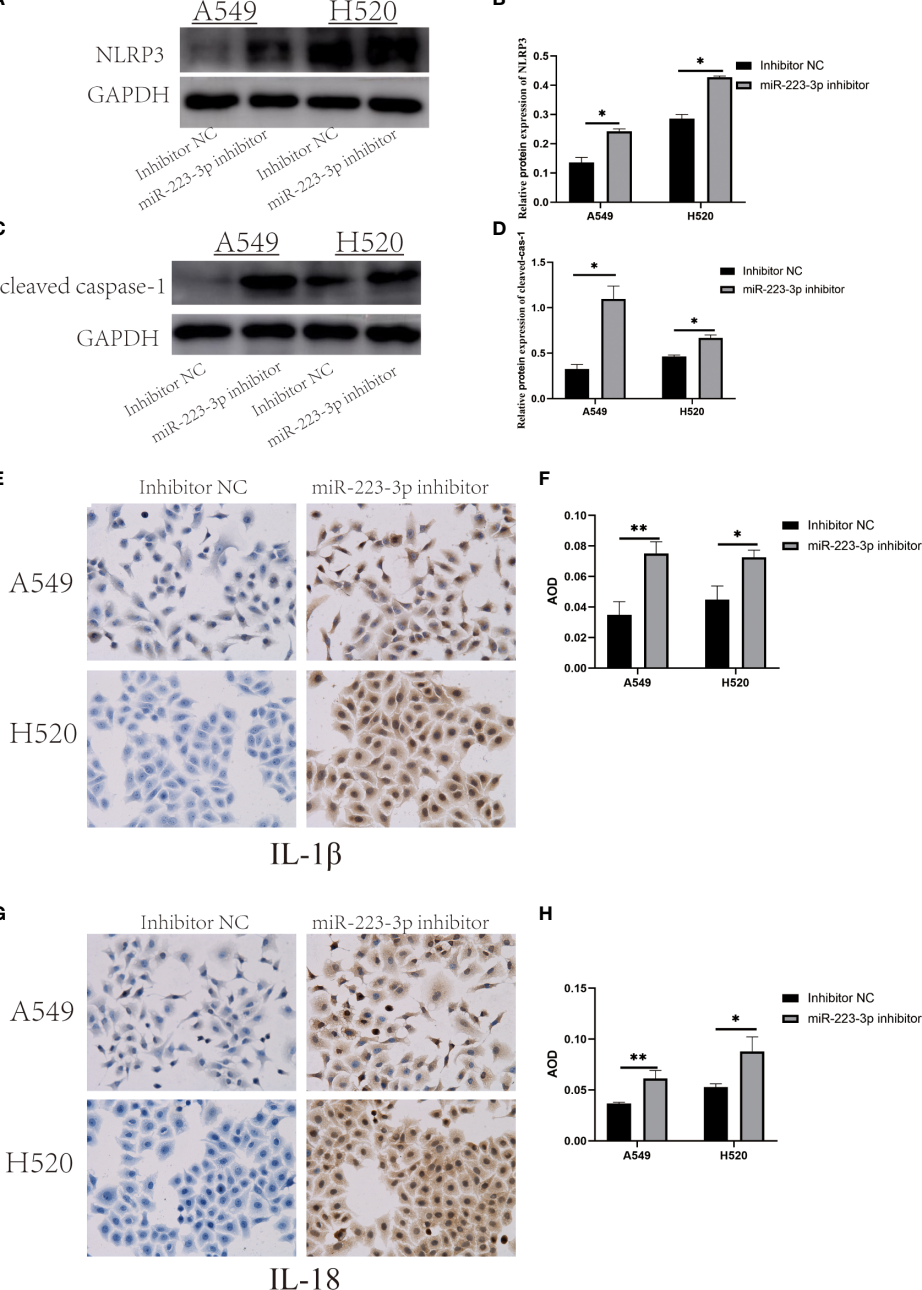
Suppression of miR-223-3p promoted the expression and activation of NLRP3 inflammasome. **(A)** The expression of NLRP3 protein were observed by Western Blot. **(B)** Quantitative analysis of NLRP3 protein were detected. **(C)** The expression of cleaved caspase-1 protein was observed by Western Blot. **(D)** Quantitative analysis of cleaved caspase-1 protein were performed. **(E)** Immunohistochemistry analysis (200×) was used to determine IL-1β protein levels. **(F)** Quantitative analysis of IL-1β protein. **(G)** Immunohistochemistry analysis (200×) was used to determine IL-18 protein levels. **(H)** Quantitative analysis of IL-18 protein. Data were presented as means ± SD of three independent experiments. *P<0.05, **P<0.01.

To assess whether miR-223-3p regulated the migration and invasion of A549 and H520 cells through the NLRP3 inflammasome regulation pathway, NLRP3 protein was firstly detected by Western Blot assay to reflect the expression of NLRP3 inflammasome. As presented in **Figures 3A, B**, NLRP3 protein was increased in A549 and H520 cells transfected with miR-223-3p inhibitor (*P*<0.05). Similar to NLRP3 protein, cleaved caspase-1 was upregulated following suppression with miR-223-3p (**Figures 3C, D**). The concentration of IL-1β and IL-18 in the inhibitor group was higher than that in the control group (**Figures 3E–H**). The results indicated that suppression of miR-223-3p promoted the expression and activation of NLRP3 inflammasome.

### Overexpression of miR-223-3p inhibited migration and invasion of A549 and H520 cells

**Figure 4:**
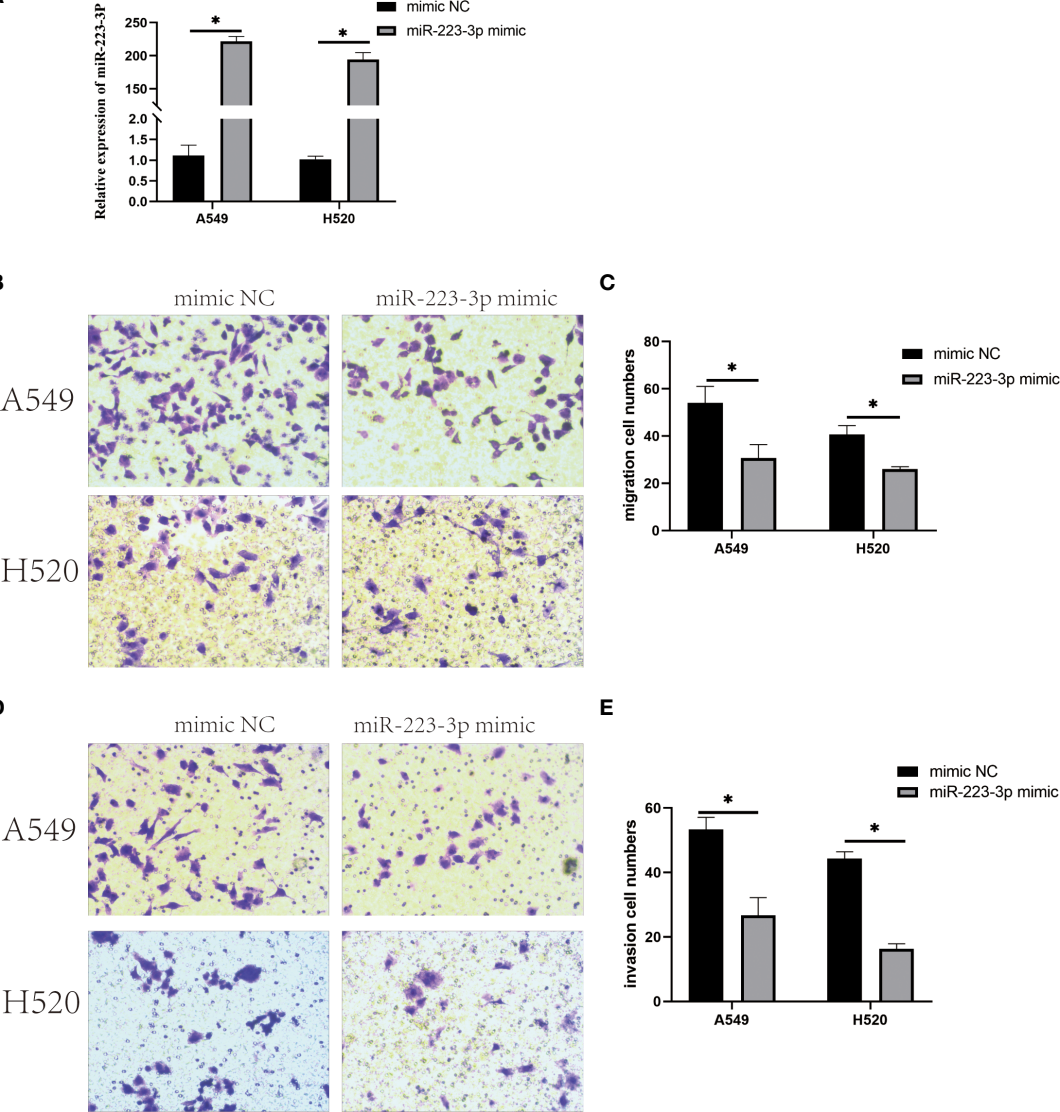
Overexpression of miR-223-3p inhibited the migration and invasion of A549 and H520 cells. **(A)** The relative expression of miR-223-3p in miR-223-3p mimic group was detected by qRT-PCR. **(B)** Cells at the bottom of the filter membrane without Matrigel matrix glue were measured in high vision field (200x). **(C)** Quantitative analysis of the migration cells of A549 and H520 were measured. **(D)** Cells at the bottom of the filter membrane with Matrigel matrix glue were detected in high vision field (200x). **(E)** Quantitative analysis of the invasion cells of A549 and H520 were measured. Data were presented as means ± SD of three independent experiments. *P<0.05.

When A549 and H520 cells were transfected with miR-223-3p mimic to overexpress miR-223-3p (**Figure 4A**), the migration and invasion were also detected by Transwell assay. From **Figures 4B, C**, we found that the average number of cells at the bottom of the filter membrane without Matrigel matrix glue was reduced in miR-223-3p mimic group comparing to the mimic NC group. Moreover, in invasion experiment, the average number of cells at the bottom of the filter membrane with Matrigel matrix glue was also reduced in miR-223-3p mimic group (**Figures 4D, E**). These results indicated that overexpressing miR-223-3p could inhibit the migration and invasion of A549 and H520 cells.

### Overexpression of miR-223-3p suppressed the expression and activation of NLRP3 inflammasome

**Figure 5:**
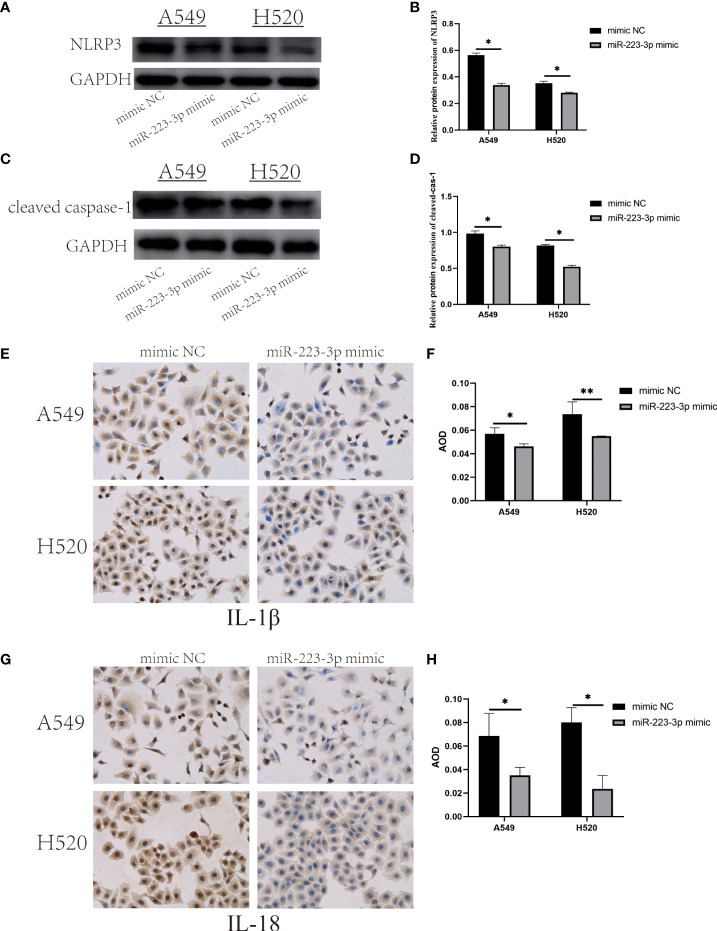
Overexpression of miR-223-3p suppressed the expression and activation of NLRP3 inflammasome. **(A)** The expression of NLRP3 protein was observed by Western Blot. **(B)** Quantitative analysis of NLRP3 protein. **(C)** The expression of cleaved caspase-1 protein was observed by Western Blot. **(D)** Quantitative analysis of cleaved caspase-1 protein. **(E)** Immunohistochemistry analysis (200×) was used to determine IL-1β protein levels. **(F)** Quantitative analysis of IL-1β protein. **(G)** Immunohistochemistry analysis (200×) was used to determine IL-18 protein levels. **(H)** Quantitative analysis of IL-18 protein. Data were presented as means ± SD of three independent experiments. *P<0.05, **P<0.01.

To measure the expression and activation of NLRP3 inflammasome in miR-223-3p mimic group. NLRP3, cleaved caspase-1, IL-1β and IL-18 were also detected respectively. After miR-223-3p was overexpressed in A549 and H520 cells, NLRP3 and cleaved caspase-1 protein were both got a decline in western blot analysis (**Figures 5A–D**). Besides, Immunohistochemistry analysis showed that IL-1β and IL-18 were also remarkably reduced after miR-223-3p overexpression (**Figures 5E–H**). These results showed that miR-223-3p suppressed the expression and activation of NLRP3 inflammasome, and the effect could be enhanced under overexpression of miR-223-3p.

## Discussion

Lung cancer is the leading cause of cancer-related death worldwide. Most lung tumors have developed distant metastasis at the time of initial diagnosis, but the regulatory mechanism of the migration and invasion has not been figured out entirely yet. Therefore, it is vital to explore the mechanism of the development of lung cancer, which will provide an important clue for the effective treatment for lung cancer.

NLRP3 is an important member of NLRs family. The role of NLRP3 in cancer cells remains controversial. The studies of Salcedo R. et al. and Takagi H. et al. showed that the NLRP3 had a protective role in colitis-associated colorectal cancer because of its ability to mediate secretion of IL-18, a cytokine which contributed to epithelial barrier repair against damage (22, 23). However, as to other cancers, such as fibrosarcoma, melanoma, gastric carcinoma, and lung cancer, NLRP3 functioned as a deleterious protein owing to its ability to suppress activation of NK cells that secrete IFN-γ and kill tumor cells (24). Besides, it was found that NLRP3 inflammasome activation could promote nicotine-induced lung cancer cell proliferation and migration (25). NLRP3 is regulated by many miRNAs. However, which miRNAs influence the migration and invasion of NSCLC through regulating NLRP3 were ill-defined. In this study, we selected the miRNAs targeting NLRP3 from TargetScan and miRDB Online Database, and they were miR-223-3p, miR-22-3p and miR-1305. Reports showed that miR-223-3p regulated the proliferation and migration of lung cancer cells by targeting the human transforming growth factor β receptor 3 (TGFBR3) (26). MiR-22-3p suppressed cell growth *via* MET/STAT3 signaling in lung cancer (27). MiR-1305 was down-regulated in NSCLC tissues and cell lines and it inhibited the progression of NSCLC cells by regulating MDM2 (28). According to the qRT-PCR results, we found the expression of miR-223-3p was upregulated in both A549 and H520 cells (**Figure 1B**). Therefore, miR-223-3p was verified further.

In the present study, the results demonstrated that the migration and invasion of A549 and H520 cells were promoted after inhibiting miR-223-3p, and the corresponding results were contrary in miR-223-3p mimic group, which suggested miR-223-3p could attenuate the migration and invasion of NSCLC cells. In addition to NSCLC, miR-223-3p influences the progression of various solid tumor types. In human osteosarcoma, miR-223-3p functioned as a tumor suppressor to inhibit the metastasis and progression of osteosarcoma through regulating Cadherin-6 (CDH6) (29). This tumor-inhibitory role of miR-223-3p was also reported in oral squamous cell carcinoma (OSCC) that miR-223-3p inhibited the proliferation and metastasis of OSCC cells by targeting SHOX2 (30). However, it was found that miR-223-3p promoted the proliferation, invasion and migration of colon cancer by negative regulating PRDM1 (31). Moreover, miR-223 was revealed to promote the invasion and metastasis of gastric cancer by regulating erythrocyte membrane protein band 4.1-like 3 (EPB41L3) (32). These studies comprehensively indicated bidirectional roles of miR-223-3p during tumorigenesis and progression.

In addition, the levels of NLRP3, cleaved caspase-1, IL-1β and IL-18 were increased in the two lung cancer cells with miR-223-3p inhibition, and the corresponding results were contrary in miR-223-3p mimic group. These results implied that miR-223-3p suppressed the migration and invasion of the two cell lines by directly regulating NLRP3. The regulation of NLRP3 by miR-223-3p was also reported in other diseases. Previous studies observed that miR-223-3p could regulate NLRP3 to promote apoptosis and inhibit proliferation of hep3B cells (33). In addition, miR-223-3p was found to influence the proliferation and migration of bladder cancer through regulating NLRP3 (34). Furthermore, dual-luciferase reporter observed that co-transfection with miR-223 reduced the luciferase activity of the plasmid containing the wild-type of the respective fragment of NLRP3 3’-UTR, while the luciferase activity of the plasmid containing the mutant NLRP3 3’-UTR fragment was not affected by co-transfection with miR-223 mimics or negative control, which indicated that miR-223 directly interacted with the 3’-UTR of NLRP3 mRNA (33). Moreover, we infer that the reason of miR-223-3p suppressing the migration and invasion of NSCLC cells by regulating NLRP3 is the reduced change of maturation and expression of IL-1β and IL-18. Both of the two cytokines were proven to have tumor-promoting effects on cancers. The studies of Saijo, Y. et al. showed that IL-1β enhanced the metastasis of lung cancer cells because of its ability to enhance angiogenesis (35). And in both murine and human breast cancer models, tumor progression was associated with elevated levels of IL-1β at primary and metastatic sites (36). IL-18 has also been proven to promote the occurrence and development of cancer by inhibiting anti-metastasis and immune surveillance mediated by NK cells and T cells (10). In gastric cancer, IL-18 induced the expression of the pro-angiogenic factor, vascular endothelial growth factor (VEGF), which finally promoted tumor growth and metastasis (37). These studies further suggested that miR-223-3p may inhibit the migration and invasion of NSCLC cells through regulating NLRP3.

In this study, we observed that miR-223-3p suppressed the pro-cancer effects of NLRP3 on the invasion and migration of NSCLC cells. And hopefully, this discover might contribute to the targeted therapy of NSCLC. To our knowledge, this is the first study to determine the role of miR-233-3p/NLRP3 axis in the migration and invasion of NSCLC. Of note, there were certain limitations in our study. Firstly, only two NSCLC cell lines, A549 and H520 cells, were used in all the experiments. Considering various kinds of cell lines of NSCLC, more experiments about other NSCLC cell lines are required in later studies to fully reveal the effects of miR-223-3p/NLRP3 axis on the progression of NSCLC. Furthermore, scientific *in vivo* experiments are also needed in further research to confirm the results in present study.

In conclusion, this research demonstrated the role of miR-223-3p in NSCLC cells and the relationship between miR-223-3p and NLRP3, revealing a novel mechanism in regulating the progression of NSCLC. This study may provide a new insight in the therapy of NSCLC in future.

## Data availability statement

The datasets presented in this study can be found in online repositories. The names of the repository/repositories and accession number(s) can be found in the article/**Supplementary Material**.

## Author contributions

FF, PS conceived and designed the study. XK performed the experiments. SZ wrote the paper. MS, MC, YL, PZ, QZ reviewed and edited the manuscript. All authors read and approved the manuscript. All authors contributed to the article and approved the submitted version.

## Funding

This work was supported by the National Natural Science Foundation of China (No. 81402712); Natural Science Foundation of Henan Province (No. 202300410457); National innovation and entrepreneurship training program for College Students (202110459056); the training grant for young teachers of Henan Province (2020GGJS011) and Zhengzhou University (JC21838046); the grant of Medical Science Research Foundation of Henan Province (No. YXKC2021031); the grant from the Department of Education of Henan Province, China (No. 20B330004 and 20B320042); the Project from China National Tobacco Corporation (No. 110202102015); and the scientific research program of innovation platform in State Tobacco Monopoly Administration (No. 312021AW0420).The authors declare that this study received funding from China National Tobacco Corporation and State Tobacco Monopoly Administration. The funder was not involved in the study design, collection, analysis, interpretation of data, the writing of this article or the decision to submit it for publication.

## Conflict of interest

The authors declare that the research was conducted in the absence of any commercial or financial relationships that could be construed as a potential conflict of interest.

## Publisher’s note

All claims expressed in this article are solely those of the authors and do not necessarily represent those of their affiliated organizations, or those of the publisher, the editors and the reviewers. Any product that may be evaluated in this article, or claim that may be made by its manufacturer, is not guaranteed or endorsed by the publisher.
