# Supplementary material for: MiR-223-3p attenuates the migration and invasion of NSCLC cells by regulating NLRP3
Source: Front Oncol. 2022 Oct 6;12:985962. doi: 10.3389/fonc.2022.985962 (PMC9583869; doi:10.3389/fonc.2022.985962)
Supplement: Supplementary file 1 [file DataSheet_1.docx]

Table a. The analysis of NLRP3 mRNA expression in lung cancer tissue with and without metastasis

| Group | With metastasis(n) | Without metastasis(n) | Log_2_FC* | FDR* |
| --- | --- | --- | --- | --- |
| LUSC | 7 | 411 | -0.4746 | 0.7823 |
| LUAD | 25 | 347 | -0.7933 | **0.0184** |
| Lung cancer | 32 | 758 | -0.5275 | 0.1028 |

Note: *FDR: adjusted *P* value, FDR<0.05 means significant difference. *Log_2_FC < 0: mRNA expression in tissue without metastasis > in tissue with metastasis, Log_2_FC > 0: mRNA expression in tissue without metastasis < in tissue with metastasis.

**
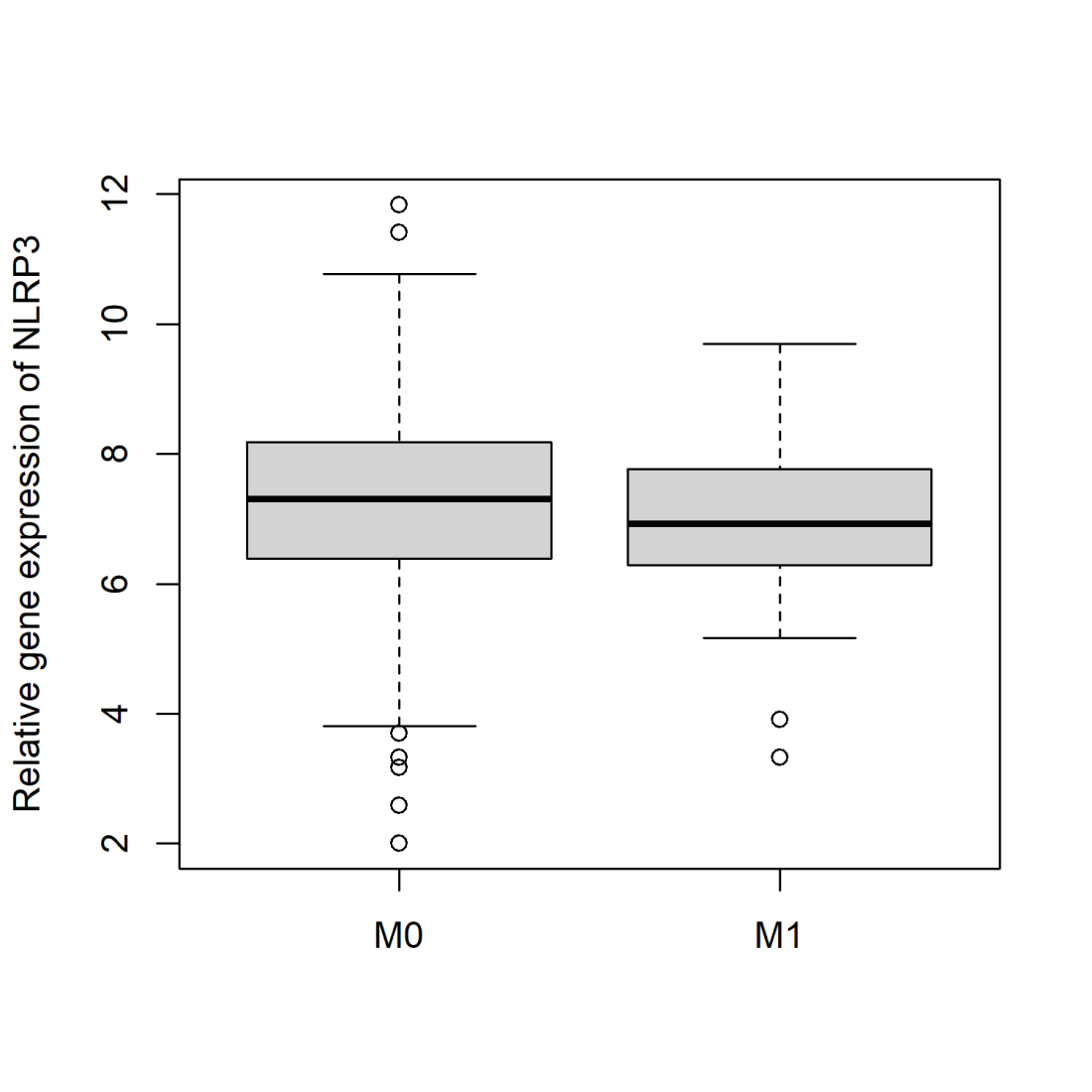
**

**Figure a.** NLRP3 mRNA expression in lung cancer tissue with and without metastasis. M0: Lung cancer patients without metastases. M1: Lung cancer patients with metastases. There was no significant difference between the tissue of lung cancer with/without metastasis (FDR=0.1028).


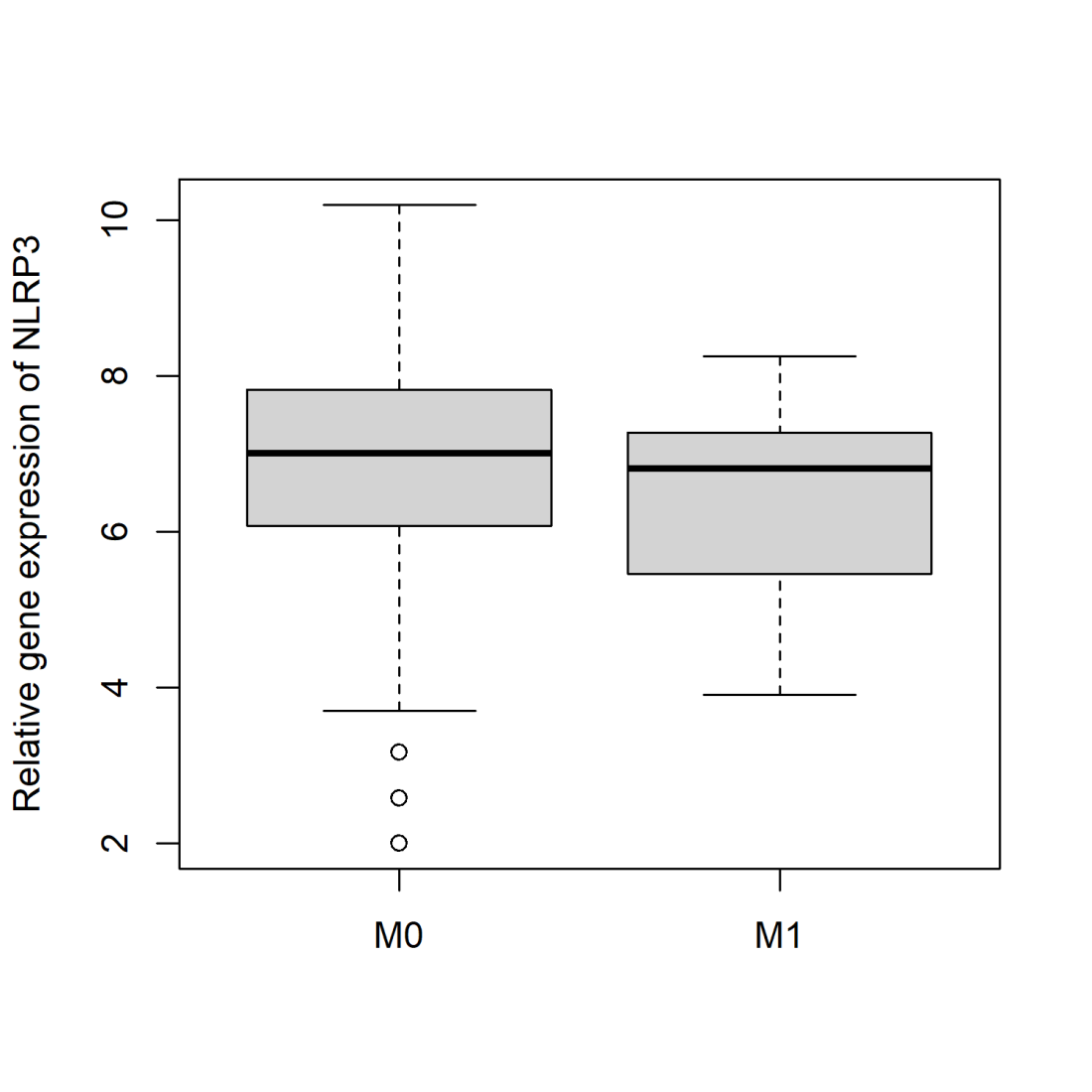


**Figure b**. NLRP3 mRNA expression in Lung squamous cell carcinoma (LUSC) tissue with and without metastasis. M0: LUSC patients without metastases. M1: LUSC patients with metastases. There was no significant difference between the tissue of LUSC with/without metastasis (FDR=0.7823).


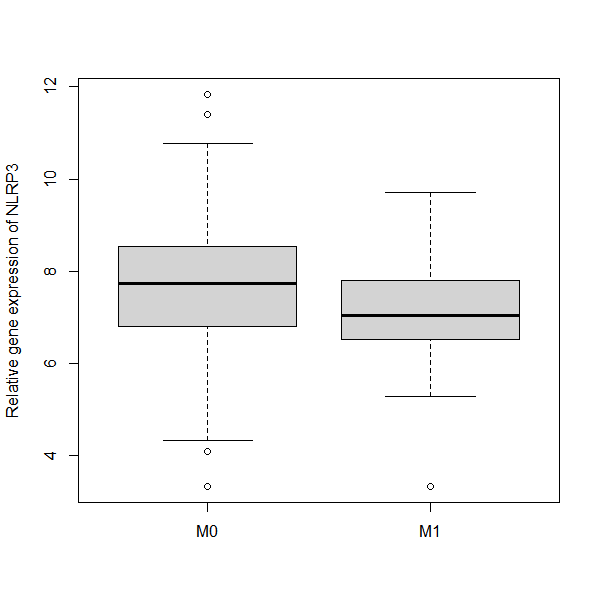
 **Figure c.** NLRP3 mRNA expression in Lung Adenocarcinoma (LUAD) tissue with and without metastasis. M0: LUAD patients without metastases. M1: LUAD patients with metastases. NLRP3 mRNA expression was significantly decreased in LUAD tissue with metastasis compared to that in LUAD tissue without metastasis (FDR =0.0184 < 0.05).
